# Supplementary material for: Comprehensive transcriptome analysis of reference genes for fruit development of Euscaphis konishii
Source: PeerJ. 2020 Feb 11;8:e8474. doi: 10.7717/peerj.8474 (PMC7020815; doi:10.7717/peerj.8474)
Supplement: Supplemental Information 1 [file peerj-08-8474-s001.pdf]

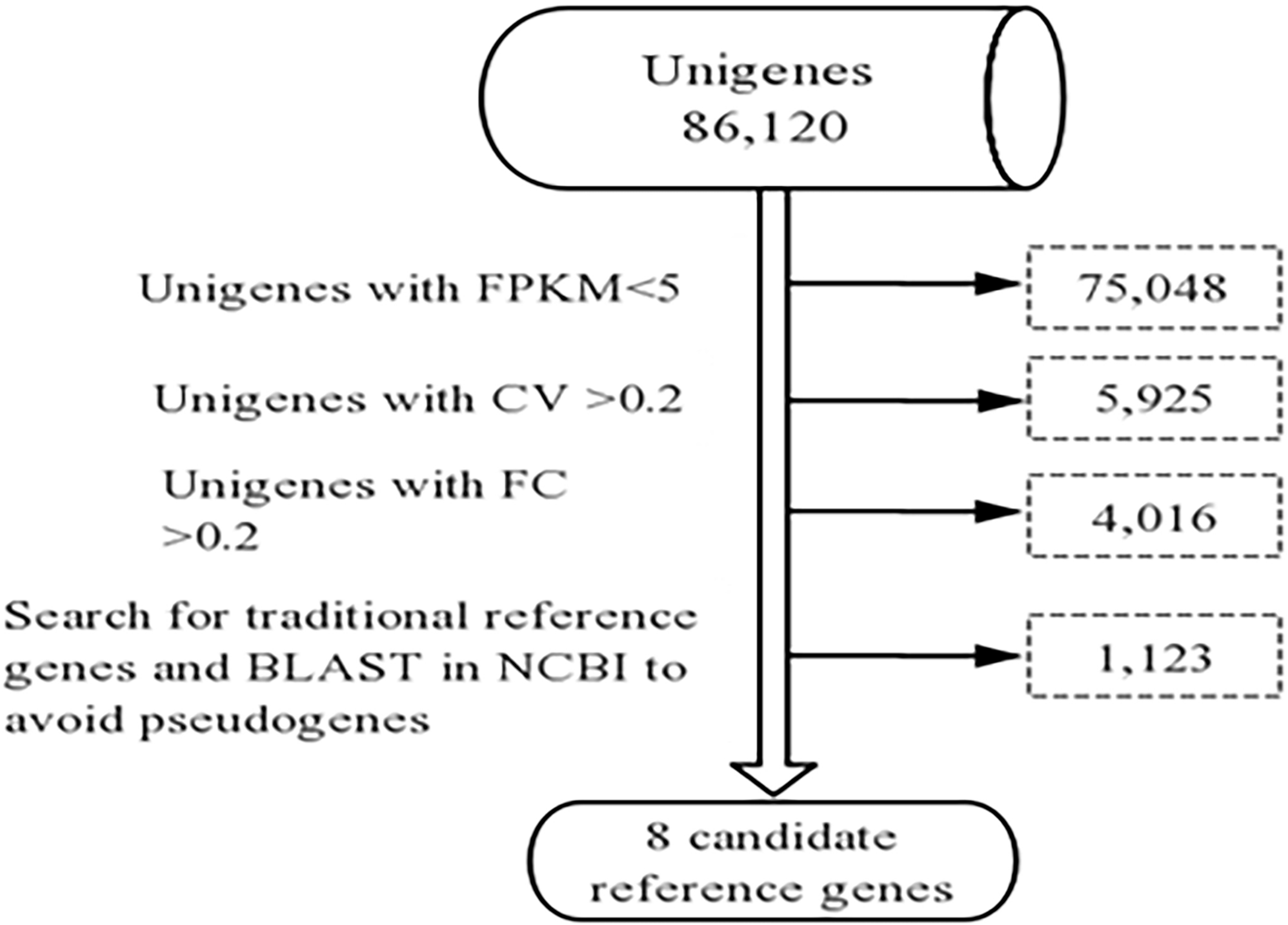
Unigenes
86,120
Unigenes with FPKM<5
75,048
Unigenes with CV >0.2
5,925
Unigenes with FC >0.2
4,016
Search for traditional reference genes and BLAST in NCBI to avoid pseudogenes
1,123
8 candidate reference genes
