# Supplementary material for: Comprehensive transcriptome analysis of reference genes for fruit development of Euscaphis konishii
Source: PeerJ. 2020 Feb 11;8:e8474. doi: 10.7717/peerj.8474 (PMC7020815; doi:10.7717/peerj.8474)
Supplement: Supplemental Information 2 — The position of primers was denoted by arrow marker symbol. [file peerj-08-8474-s002.pdf]

Protein Coding Gene Models

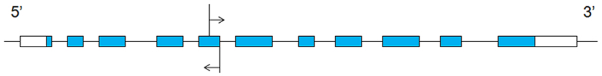

Gene ID: c71660.graph_c1

Reference gene model: AT1G13440.1(*Arabidopsis*)

Protein Coding Gene Models

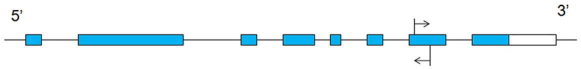

Gene ID: c67010.graph_c0

Reference gene model: AT2G16920.1(*Arabidopsis*)

Protein Coding Gene Models

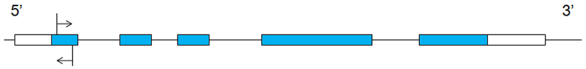

Gene ID: c67439.graph_c2

Reference gene model: AT5G19770.1(*Arabidopsis*)

Protein Coding Gene Models

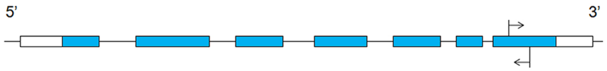

Gene ID: c67539.graph_c0

Reference gene model: AT3G01480.1(*Arabidopsis*)

Protein Coding Gene Models

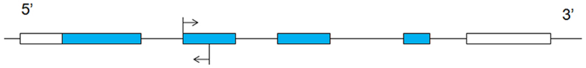

Gene ID: c62586.graph_c0

Reference gene model: AT3G52590.1(*Arabidopsis*)

Protein Coding Gene Models

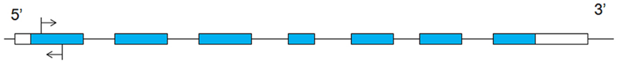

Gene ID: c65728.graph_c0

Reference gene model: AT3G15020.2(*Arabidopsis*)

Protein Coding Gene Models

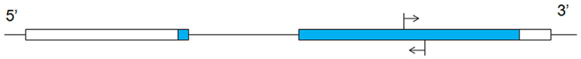

Gene ID: c63030.graph_c0

Reference gene model: AT3G47520.1(*Arabidopsis*)

Protein Coding Gene Models

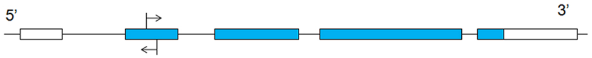

Gene ID: c63658.graph_c0

Reference gene model: AT5G09810.1(*Arabidopsis*)
