# Supplementary figures and images for: Comprehensive transcriptome analysis of reference genes for fruit development of Euscaphis konishii
Source: PeerJ. 2020 Feb 11;8:e8474. doi: 10.7717/peerj.8474 (PMC7020815; doi:10.7717/peerj.8474)

Derivative Reporter(-Rn)

*EkGAPDH2*

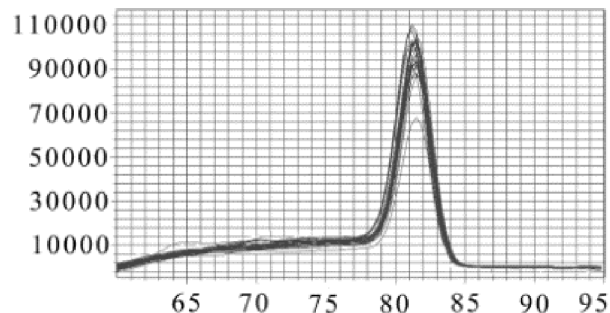

*EkTUA3*

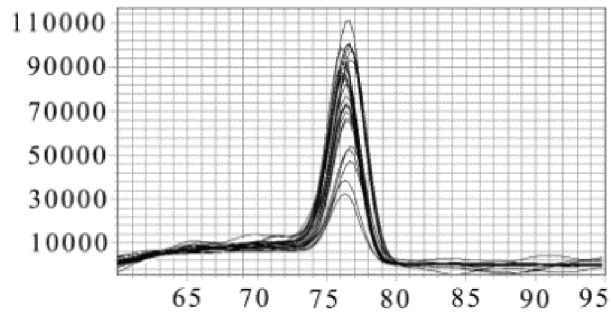

*EkCYP38*

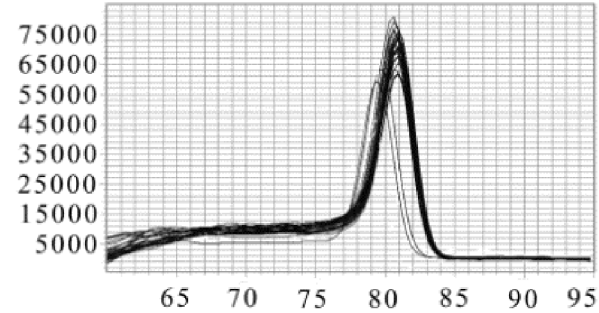

*EkUBC23*

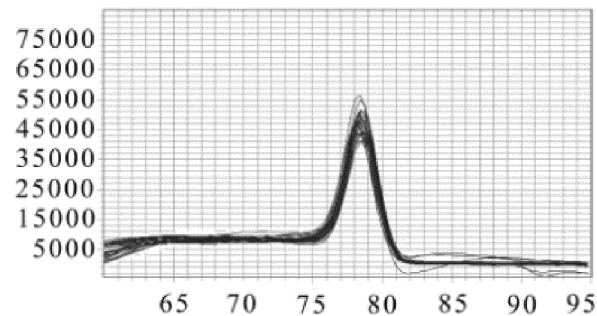

*EkUBQ1*

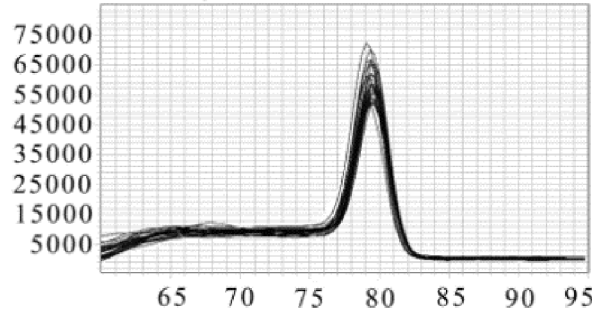

*EkMDH2*

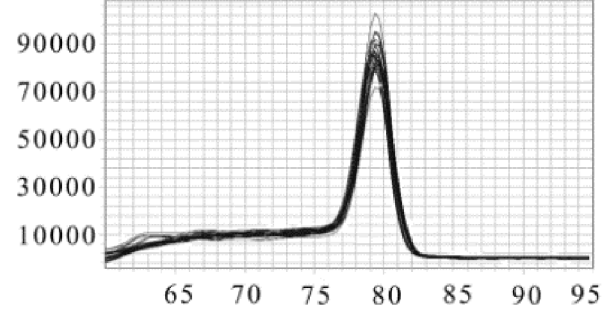

*EkMDH*

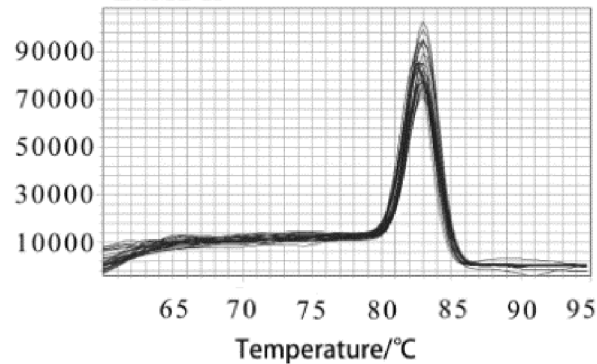

*EkACT7*

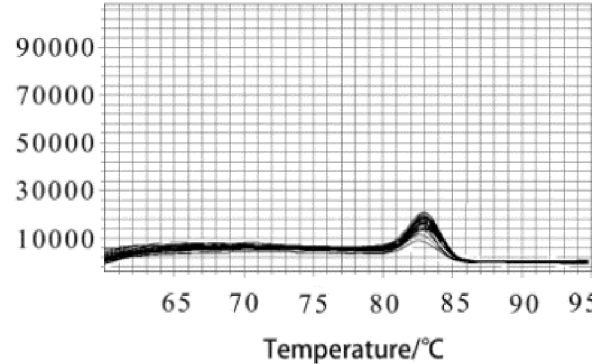

Temperature/°C

Supplement: Supplemental Information 3 — The Derivative Reporter (X-axis) was plotted versus the reaction temperature of qRT-PCR (Y-axis). A single peak of the melting curve in qRT-PCR were used to ensure the specificity of the primers for the candidate reference genes. [file peerj-08-8474-s003.pdf]

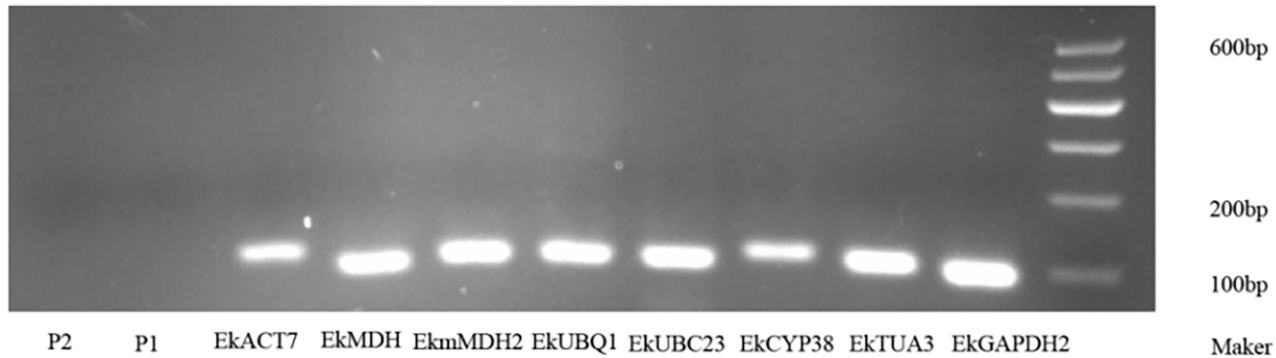

Supplement: Supplemental Information 4 — PCR products on 1.2% agarose gel stained with GoldView. Lanes 1, 2, 3, 4, 5, 6, 7 and 8 were the gene products of EkACT7, EkMDH, EkmMDH2, EkUBQ1, EkUBC23, EkCYP38, EkTUA3 and EkGAPDH2, respectively. [file peerj-08-8474-s004.pdf]

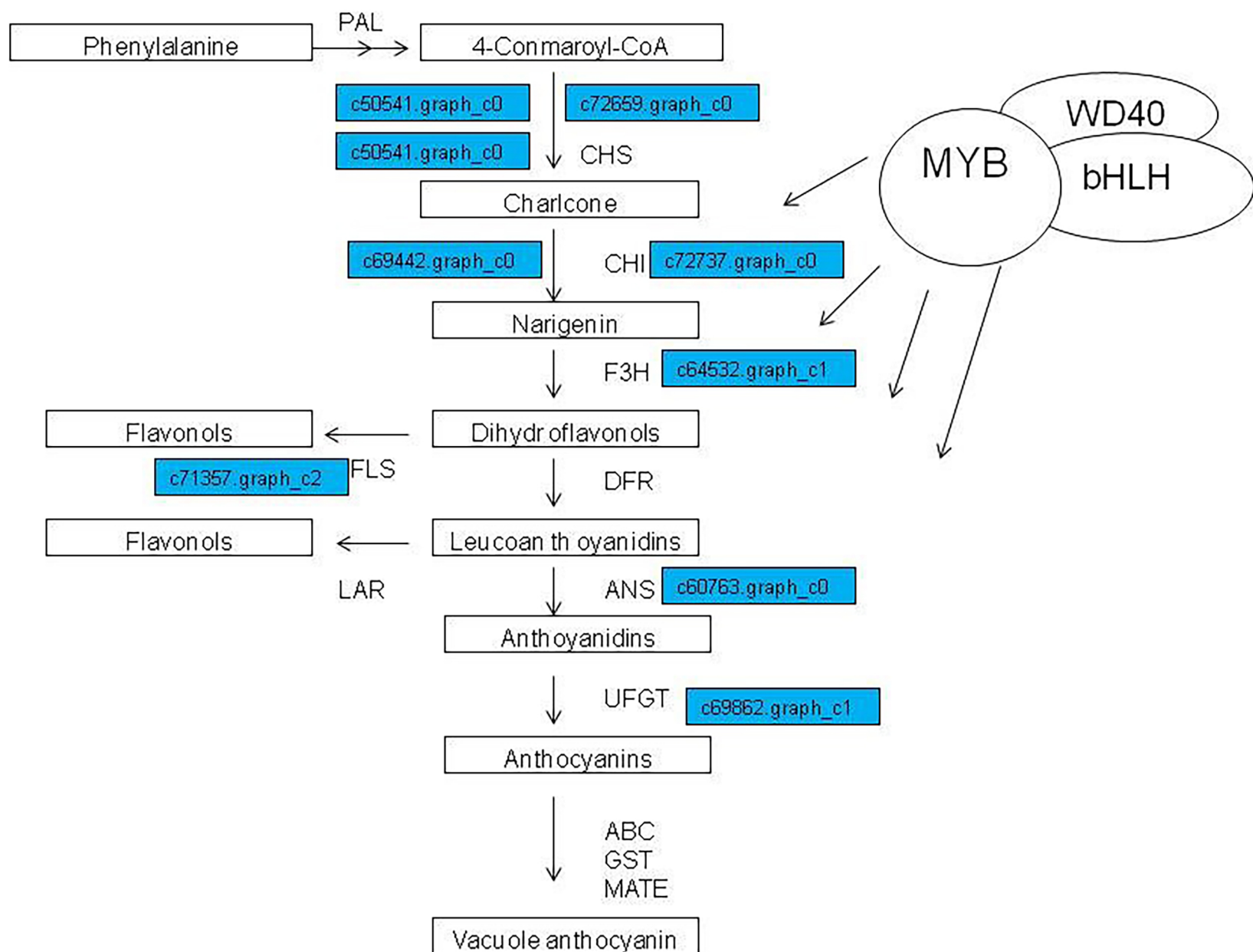

Supplement: Supplemental Information 5 — The study of our lab colleague Yuan was used as a reference that publish in International journal of molecular science: Sequencing of E. konishii endocarp transcriptome points to molecular mechanisms of endocarp coloration. The blue box indicated 9 genes related to the anthocyanin synthesis pathway. [file peerj-08-8474-s005.pdf]

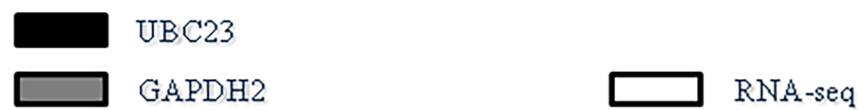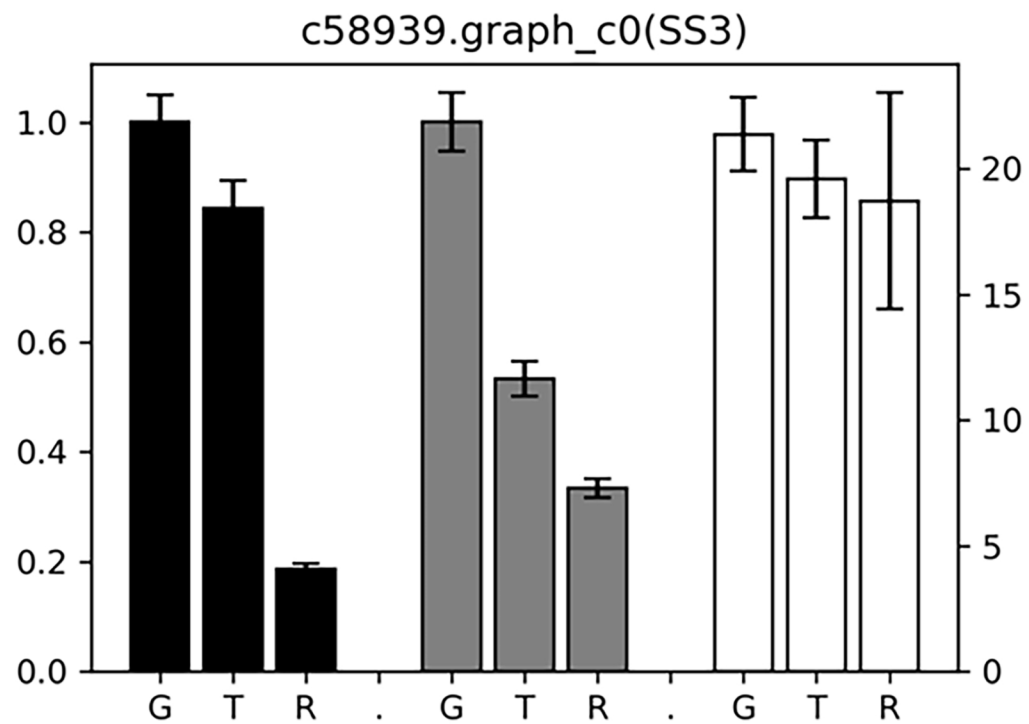

Supplement: Supplemental Information 6 — UBC23 and GAPDH2 were used to normalized EkSS3, respectively. Error bars represent standard deviation from theree independent biological replicates. “G”, “T” and “R” mean green stage, turing stage and red stage in furit, respectively. [file peerj-08-8474-s006.pdf]

**A**

1000

750

500

100

Marker

Root

Leaf

Branch

Fruit

**B**

1000

750

500

100

Marker

Root

Leaf

Branch

Fruit

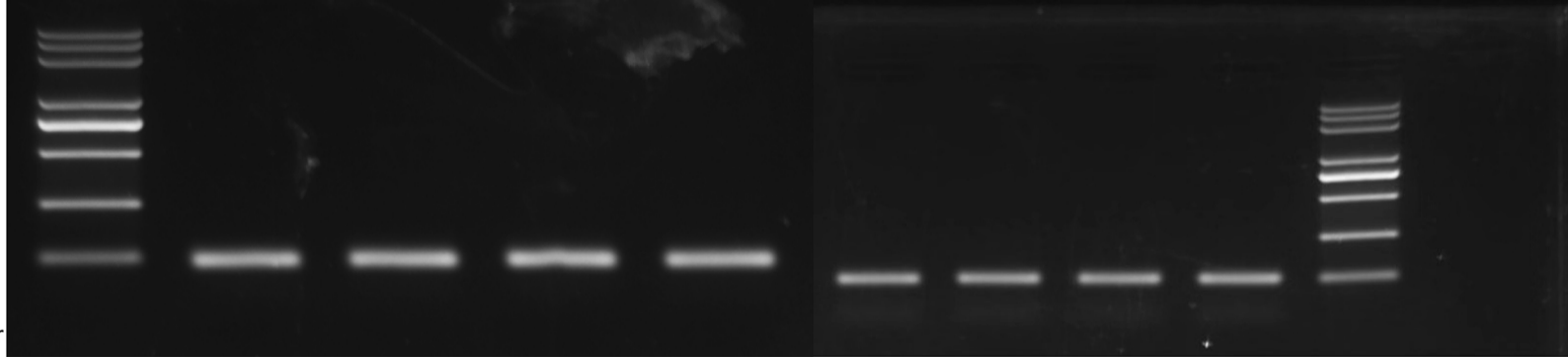

Supplement: Supplemental Information 7 — (A) The gene expression pattern of EkGAPDH2 in root, leaf, branch and fruit. (B) The gene expression pattern of EkUBC23 in root, leaf, branch and fruit. [file peerj-08-8474-s007.pdf]
