# Supplementary material for: Comprehensive transcriptome analysis of reference genes for fruit development of Euscaphis konishii
Source: PeerJ. 2020 Feb 11;8:e8474. doi: 10.7717/peerj.8474 (PMC7020815; doi:10.7717/peerj.8474)
Supplement: Supplemental Information 10 [file peerj-08-8474-s010.doc]

*> Ek**GAPDH2*

CCGTGTTCCTACTGTTGATGTCTCGGTGGTTGACCTCACTGTGAGGTTGGAGAAGGAGGCTACCTACGAGGATATTAAGGCTGCTATCAAGGAGG

*> EkTUA3*

GGGTGGTAGCAAACCCTATTACATTGAACCAATCTTCAGATTAATGCTCTAGCACTATGATATCAATAATCTTCTCCTTCCTCTTCATCATCTGCACCTTCGG

*> EkCYP38*

ATCTGTTGGAACTCCTCCATTCCAGCTTCCAGCTTGTCAAGCAGTTCCACTCCATGGTCCTTCTTTGATTCAGCAAGCCCAGATATAATTAAAGCTTTACCTTGCTTCAGGGCT

*> EkUBC23*

AGCCACATAATCTCCGTGTAAGAATCCTCGATCAACAACTGTTATATCGTTCAGTTTTTGGGTGGATTCAGTGTCATCAATCCAAACTACTCGAACATGGTCAGC

*> EkUBQ1*

ACGAGCCAAAGCCATCAAAGAAGGTTCAATAATTCCTCCGCGAAGCCTCAACACAAGATGAAGCGTTGATTCTTTCTGGATATTGTAATCAGCAAGAGTTCGGCC

*> EkmMDH2*

CATCGTAAGTCCCTGCTTTCTTGAAAACCTCAGCAGCAATTGGAACTGTCGAGTTCACAGGGTTGCTTATCATATTAACAAGAGCATTAGGGCAGTACTTGGCA

*> EkMDH*

ATGAAGAAGTCCACGAGCTAACTGCTAGGATCCAAAATGCTGGGACAGAAGTTGTGGAGGCGAAGGCTGGTGCAGGTTCTGCTACTCTGTCTATGGC

*> EkACT7*

GATCTGGCATCACACCTTCTACAATGAGCTTCGAGTTGCTCCTGAGGAGCACCCAGTACTTCTCACAGAGGCACCTCTCAACCCTAAGGCCAACAGGGAGAAGATGACTCAG
